# Mixed Matrix Membranes Using Porous Organic Polymers (POPs)—Influence of Textural Properties on CO_2_/CH_4_ Separation

**DOI:** 10.3390/polym15204135

**Published:** 2023-10-18

**Authors:** Laura Matesanz-Niño, Jorge Moranchel-Pérez, Cristina Álvarez, Ángel E. Lozano, Clara Casado-Coterillo

**Affiliations:** 1 Department of Applied Macromolecular Chemistry, Instituto de Ciencia y Tecnología de Polímeros, ICTP-CSIC, Juan de la Cierva 3, E-28006 Madrid, Spain; laura.matesanz@ictp.csic.es (L.M.-N.); cristina.alvarez@ictp.csic.es (C.Á.); lozano@ictp.csic.es (Á.E.L.); 2 Surfaces and Porous Materials (SMAP, UA-UVA_CSIC), Associated Research Unit to CSIC, University of Valladolid, Paseo Belén 7, E-47011 Valladolid, Spain; 3 Department of Chemical and Biomolecular Engineering, Universidad de Cantabria, E-39005 Santander, Spain; jorge.moranchel@alumnos.unican.es; 4 IU CINQUIMA/Química Inorgánica, Facultad de Ciencias, Universidad de Valladolid, E-47071 Valladolid, Spain

**Keywords:** gas separation, Matrimid, Pebax, biopolymers, mixed matrix membranes (MMMs), porous organic polymers (POPs), CO_2_/CH_4_ separation, Maxwell phenomenological equations

## Abstract

Mixed matrix membranes (MMMs) provide the opportunity to test new porous materials in challenging applications. A series of low-cost porous organic polymer (POPs) networks, possessing tunable porosity and high CO_2_ uptake, has been obtained by aromatic electrophilic substitution reactions of biphenyl, 9,10-dihydro-9,10-dimethyl-9,10-ethanoanthracene (DMDHA), triptycene and 1,3,5-triphenylbenzene (135TPB) with dimethoxymethane (DMM). These materials have been characterized by FTIR, ^13^C NMR, WAXD, TGA, SEM, and CO_2_ uptake. Finally, different loadings of these POPs have been introduced into Matrimid, Pebax, and chitosan:polyvinyl alcohol blends as polymeric matrices to prepare MMMs. The CO_2_/CH_4_ separation performance of these MMMs has been evaluated by single and mixed gas permeation experiments at 4 bar and room temperature. The effect of the porosity of the porous fillers on the membrane separation behavior and the compatibility between them and the different polymer matrices on membrane design and fabrication has been studied by Maxwell model equations as a function of the gas permeability of the pure polymers, porosity, and loading of the fillers in the MMMs. Although the gas transport properties showed an increasing deviation from ideal Maxwell equation prediction with increasing porosity of the POP fillers and increasing hydrophilicity of the polymer matrices, the behavior of biopolymer-based CS:PVA MMMs approached that of Pebax-based MMMs, giving scope to not only new filler materials but also sustainable polymer choices to find a place in membrane technology.

## 1. Introduction

The present concern of the current climate emergency is stirring worldwide interest in the development of materials and technologies for the decarbonization of industry and society by CO_2_ capture, clean energy production, and biogas upgrading. Biogas upgrading offers the possibility to recover both methane and carbon dioxide for ulterior utilization [1]. Membrane technology for the simultaneous recovery of CO_2_ and CH_4_ fluxes from different sources will play a key role in the development of industrial materials with higher efficiency than current ones [2,3,4]. Commercially available membranes have performed well in CO_2_/CH_4_ separation on the pilot scale, as Sepuran^®^ [5], Polyactive™ [6], and Polaris^®^ [7]. The well-known trade-off between permeability and selectivity in polymeric membranes has been a major drawback for the larger deployment of membrane technology in CO_2_ separation applications, together with the issue of uncertainty regarding stability in real industrial feed gas streams [8,9,10], leading to the development of new materials to face the challenges of existing ones. Most of the studied materials in CO_2_/CH_4_ separation are based on polyimides [11] and block co-polymers [12]. Most recently, the focus has been turned to biopolymers and materials from renewable sources [13,14] such as chitosan [15].

Attempts have been made to overcome these issues by well-designed mixed matrix membrane material (MMMs), involving the loading of polymer matrices with small amounts of advanced fillers to obtain synergistic properties between the two components [16]. Interfacial control in mixed matrix membranes has always been an issue to be solved. This point has been addressed by the outcome of porous organic materials, or COMs, selective for the gases of interest, as fillers into polymeric membranes, because their organic nature allows the expectation of higher selectivity than zeolite or metal oxide fillers. Porous covalent–organic materials (COMs) have high surface areas and diverse pore dimensions, topologies, and chemical functionalities, for which they are attracting interest in a range of scientific fields, from gas storage to energy applications [17]. Porous polymer networks (PPNs) offer high free volume and CO_2_ uptake [18]. There are several good perspectives, reviews, and research articles on different kinds of such organic porous fillers, from metal organic frameworks [19] and covalent organic frameworks [20] to porous organic cages (POCs) [21], amorphous scrambled derivatives (ASPOCs) [22], porous organic frameworks (POFs) blended with polysulfone [23], supramolecular organic frameworks combined with Matrimid [24], and hydrogen-bonded porous polymers blended with Pebax 1657MH [25]. Note that most of the studies are focused on the characterization of the POP particles and their effect on improving the permselectivity of conventionally not highly permeable or selective well-known polymers. Most of these studies, as reflected in Table 1, are dealing with the synthesis and characterization of the materials. The only hint about the gas separation performance is given by time-lag single gas permeation measurements in a constant volume setup. MMMs were prepared on commercial polymers with low permeability and/or selectivity such as Matrimid and Pebax, to evaluate the influence of the loading of the new porous fillers. As far as we know, only Gao et al. [23] used 50:50 (*v*/*v*%) binary gas mixtures of CO_2_/CH_4_ and CO_2_/N_2_ to characterize the separation performance. More recently, Wang et al. studied the effect of humidity in mixed gas separation performance, at a concentration of 30:70 (*v*/*v*%) for the case of CO_2_/CH_4_ separation [25].

The understanding of this interaction has been modeled with several phenomenological model approaches based on the Maxwell equation, enabling the correlation between the permeability of novel membrane materials from the components of the blend or mixture. Recent reviews have shed light on research efforts that account for non-idealities in the behavior of Maxwell’s equation for different MMMs, through attempts to quantify chain rigidification and interfacial distances between the dispersed filler and the polymer continuous phases [26], as a function of the volume fraction and dispersion of the porous filler and the permeability of the gas components through the dispersed and continuous phases. The understanding of the CO_2_ separation performance of MMMs filled with porous organic networks has also been envisaged by the Maxwell equation, for MWNTs in Pebax [27], knitting aryl polymers, KAPs, in polycarbonate [28], and imine/imide porous organic cages in Matrimid [21]. Recently, a hydrophobic amorphous porous organic polymer (POP-2) containing triarylamines linked by 1,4-diethynylphenyl bridges was compared as filler to Matrimid with metal–organic frameworks such as ZIF-8 and Cu-BTC, regarding the CO_2_ permeability in the presence of H_2_S impurities [29]. However, the lack of sufficient experimental data on the permeability of CO_2_ and CH_4_ through the porous organic polymer dispersed phases in MMMs, makes necessary the definition of parameters to estimate the membrane design requirements for a certain CO_2_/CH_4_ separation. Minelli et al. [30] use the ratio α, i.e., the permeability ratio between the dispersed and continuous phases to be able to compare the Maxwell model equation considering different morphologies and interaction between the phases: (i) parallel orientation of the particles to the direction of the flux, (ii) normal or in series, (iii) Maxwell model for spherical particles, and (iv) Wiener’s equation introducing the different shape factor or the dispersed phase within the continuous matrix. These phenomenological model approaches fail to describe the large differences observed in MMMs when highly porous fillers, such as carbon molecular sieves (CMS) and ad-hoc modification consisting of applying these model equations twice to account for the interface between the particle and the polymer matrices, were proposed to predict the non-ideal performance of polyimide-filled MMMs [31]. The development of complex polymer matrices by co-polymerization [32,33] and the recent attention of researchers on the potential of bio-based polymers and fillers in membrane separation makes room for unexplored non-idealities worth understanding [13,34,35].

**Table 1:** Mixed matrix membranes reported from porous organic polymer networks for CO_2_/CH_4_ separation.

Porous Organic Filler	Polymer Matrix	Filler Loading (wt.%)	P(CO_2_) (Barrer)	CO_2_/CH_4_ Selectivity	Other Characterization
Pillar [5] arene, SOF [24]	Matrimid 5218™	0	73 ± 2	27 ± 5	Single gas permeation, 20 °C, 1 atm PXRD, SEM
		10	63 ± 4	31 ± 7	
		50	75 ± 4	25 ± 4	
POP2 [29]	Matrimid 5218	20	26.9 ± 1.0	35.86	Pure gas permeability in the absence and presence of H_2_S in CH_4_ and/or N_2_
POCs [21]	Matrimid 9725 PEEK-WC	0	10.8	31.1	Single gas permeation ^1^H NMR, SEM, PXRD, SXRD, ATR-FTIR, TGA, gas sorption at 273 K and 25 °C, BET
		20	16.7	41.7	
		0	6.04	23.9	
		20	6.15	25.7	
SNW-1 [36]	Polysulfone (PSf)	0 12	8.00 22.4	17.5 34	Mixed gas CO_2_:CH_4_ (1:1) Permeation, 298 K ^13^C CP/MAS NMR, ^15^N CP/MS NMR, TGA, mechanical properties, SEM, FTIR, BET
HOF-21 [25]	Pebax MH 1657	0	240	8	FTIR, ^13^C NMR, SEM-EDX, TGA, PXRD, DFT
		3	780	40	

In a previous work, a POP material derived from 4,5-diazafluoren-9-one (DAFO) and 1,3,5-triphenylbenzene (135TPB) containing bipyridine functionality was characterized as filler in Matrimid mixed matrix membranes regarding single gas permeability of CO_2_, CH_4_ and N_2_, and olefin/paraffin separation [37]. In this work, five different hyper-crosslinked porous organic polymers (POPs) synthesized at the Institute of Polymer Science and Technology (ICTP-CSIC) were added as dispersed fillers to three different polymers in CO_2_ separation as continuous matrices, in order to evaluate the potential of novel materials in CO_2_ separations. The effect of porosity and CO_2_ uptake properties of the POPs on the permeability and selectivity of the resulting membranes was assessed. The polymers chosen for the continuous matrix were Pebax (60%/40%) and a 50:50 *v*/*v*% blend of biopolymer chitosan (CS) and biodegradable low-cost polymer polyvinyl alcohol (PVA), in quest of the circularity of membrane preparation [13]. The performance of the membranes was analyzed by mixed CO_2_/CH_4_ gas separation and compared with previous work where a POP filler similarly containing bipyridine functionality was used as filler in Matrimid MMMs, regarding the single gas permeability of CO_2_, CH_4_ and N_2_, and olefin/paraffin separation [37]. The performance of the gas separation was analyzed in terms of phenomenological model equations to evaluate the deviations from previously reported MMM behavior [38].

## 2. Materials and Methods

### 2.1. Materials

Matrimid 5218 (made from 3,3′,4,4′-benzophenone tetracarboxylic dianhydride and diaminophenylindane) was supplied by Hunstman (Merrimack, NH, USA). Pebax^®^ 1657 MH was supplied by Arkema (Colombes, France). Chitosan (CS) was purchased from Sigma Aldrich (deacetylated degree 75% and molecular weight 310,000 to 375,000). Polyvinyl alcohol (PVA, 99+% hydrolyzed, with a molecular weight 85,000 to 124,000 g/mol) was also purchased by Sigma Aldrich (Madrid, Spain).

### 2.2. Synthesis Procedures

**Figure 1:**
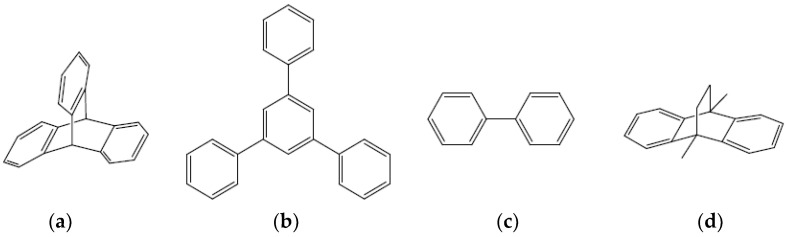
Bifunctional and trifunctional aromatic molecule constituents of the POP structure: (**a**) triptycene, (**b**) 135-TPB, (**c**) biphenyl, (**d**) DMDHA.

The POPs were synthesized by the reaction of aromatic trifunctional symmetric monomers (triptycene and 1,3,5-triphenylbenzene, 135-TPB) separately, together, or co-polymerized with bifunctional aromatic monomers, as biphenyl and 9,10-dihydro-9,10-dimethyl-9,10-ethaneantracene, DMDHA, using dimethoxymethane (DMM) as a linker promoter in the presence of a Lewis acid catalyst (FeCl_3_). Table 2 collects the composition of the POPs prepared for this work and the initial molar proportion of monomers and starting reactants from the aromatic molecules whose chemical formula is represented in Figure 1. The solvent dichloroethane was added in 15–30 times volume to the mmol of DMM (v/mol). The reaction temperature was set to 60 °C for 72 h.

#### Synthesis of Polymer Membranes

Membranes were prepared by adding different filler loadings from 0 to 10 wt.% to the total polymer content of the different POPs in different polymers whose main physical properties are collected in Table 3.

Matrimid membranes were prepared as reported elsewhere [37], by dissolving 450 mg Matrimid in 10 mL chloroform (Scharlau) under magnetic stirring for 24 h at room temperature. The solution was then poured on a leveled glass plate kept at room temperature, limited by a glass ring to obtain a homogeneously thick film. To avoid fast evaporation of the solvent, the ring was slightly covered with a glass funnel. The film resulting after solvent evaporation was removed from the glass plate and treated in a vacuum oven (Heraeus Vacutherm) at 60 °C (90 min), 120 °C (120 min), 150 °C (60 min), and 220 °C (60 min) and cooled down slowly in the oven. The average membrane thickness was 50 ± 3 µm.

Pebax membranes were prepared by dissolving 3 wt.% Pebax in a 70:30 *v*/*v*% ethanol/water mixture at 90 °C for 6 h, then removing the bubbles if needed by using an ultrasound bath for 10 min, and casting on hydrophobized glass Petri dishes of 4.5 cm diameter. The solvent was evaporated in a fume hood for 24–48 h, slightly covered at room temperature, then dried in a vacuum at 40 °C to a constant weight, and removed from the glass plate.

CS:PVA membranes were prepared as reported elsewhere [48], from equivalent volumetric blends of CS 1 wt.% solution in 2 wt.% aqueous acetic acid solution and PVA 4 wt.% aqueous solutions prepared independently by stirring at room temperature and under reflux at 80 °C for 24 h before blending. The membranes were likewise cast in Petri dishes and the solvent evaporated for 2–3 days at room temperature in a fume hood and neutralized by immersion in NaOH 1 M solutions for 1 h, then rinsed with DI water to be removed from the glass [49,50].

Mixed matrix membranes were prepared by adding polymer solutions to the suspension of the POP material in 2 mL of the corresponding solvent. In the case of the Matrimid-based MMM, it was necessary to sonicate the suspension for 20 min before casting, to avoid agglomerates [37]. In the case of Pebax-based MMMs, the POPs were previously treated with air-based low-pressure plasma (Piezo brush^®^ PZ3, Reylon plasma, Regensburg, Germany) for 30 s to hydrophilize the surface [51] and ease the compatibility with the Pebax matrix. In the case of Pebax-based MMMs, the POPs were previously treated with air-based low-pressure plasma (Piezo brush^®^ PZ3, Reylon plasma) for 30 s to hydrophilize the surface [51] and ease the compatibility with the Pebax matrix. This was not necessary for the CS:PVA membranes, which was attributed to the high hydrophilicity of the biopolymers, which compensated for the differences between the dispersed and continuous phases in the membrane matrix. The particle loadings of POP in the MMMs were calculated as

(1) $$\emptyset_{d}=\frac{weight\;of\;particle}{\left(weight\;of\;particle+weight\;of\;polymer\right)}\times 100$$

### 2.3. Characterization

ATR–FTIR experiments were conducted on the POP and membrane samples using a Spectrum 65 Spectrophotometer (Perkin Elmer, Waltham, MA, USA) at a 4 cm^−1^ resolution and 8 scans per measurement, in the range of wave numbers of 4000–400 cm^−1^.

^13^C NMR of the POPs were registered in a solid-state Avance TM 400 WB (Bruker, Mannheim, Germany), equipped with a superconductor wide magnet (89 mm) operating at 9.4T, using cross-polarization (CP) and magic angle spinning (MAS). The spectra were registered at a frequency of 100.6 MHz and contact pulses of 1 ms, with a delay time of 3 s, and a spinning speed of 11 kHz.

The WAXD diffractograms of POPs were registered at room temperature in a Bruker D8 Advance diffractometer, equipped with a Cu X-ray source (wavelength λ = 1.54 Å, a Göbel mirror, and a Vantec detector, at a step of 0.024° and a rate of 0.5 s/step, in the interval of 2θ from 3 to 60°.

SEM images were obtained using a scanning electronic microscope with field emission filament QUANTA 200 FEG ESEM, Hillsboro, OR, USA. The membrane film samples were prepared by cryogenic fracture after immersion in liquid nitrogen, and they were Au-metallized.

The skeletal density of POPs was measured in a He pycnometer (AccuPyc, Micromeritics). The density of the polymer and mixed matrix membranes was estimated from the weights and thicknesses of the circular pieces of membranes before and after gas separation experiments [47].

Adsorption/desorption isotherms of the POPs were measured in a N_2_ volumetric analyzer (ASAP2020, Micromeritics) at 77 K of the previously degassed samples at 200 °C for 16 h. The surface area was calculated from the adsorption isotherms by the Brunauer–Emmett–Teller (BET) method, and the pore volume was obtained at a relative pressure of around p/p_0_ = 0.98. The microporosity of samples was estimated by the t-plot method. CO_2_ uptake in the POP fillers was conducted in a Cahn D200 microbalance at 25 °C [37].

Thermogravimetric analyses (TGA) were realized in a TA-Q500 (TA instruments, New Castle, DE, USA) for the POPs at a heating rate of 10 °C in the interval of 30–850 °C under N_2_ (50 mL/min) whereas 1–5 mg membrane samples of the films were measured at a TGA–DTA Shimadzu (Kyoto, Japan) in the range of 25–600 °C at a heating rate of 20 °C/min, also under N_2_ flow of 50 mL/min [52].

The thickness of the MMMs was determined by a Mitutoyo IP-65 with a precision of 0.001 mm, at 5 different spots on the membrane surface area after synthesis. The standard deviation of these measurements was below 0.003 cm for all the membranes under study.

### 2.4. Gas Transport and Separation

Single gas permeability values of He, H_2_, CO_2_, N_2_, and CH_4_ gases across the neat polymer and MMMs were determined at 30 °C and a feed pressure of 3 bars, in a constant volume/variable pressure system at the ICTP–CSIC (Madrid, Spain). Before each measurement, the membrane was kept in a high vacuum overnight to remove humidity and solvent traces. The absence of pinholes was checked by He permeation, at pressures between 1 and 5 bars. The membrane was then subjected to a gas pressure of 3 bars, and the rise of permeate pressure (gas through the membrane) was monitored as a function of time until a steady stationary state was attained, where the relationship between permeate pressure and time was linear.

The permeability of the membrane, P, in steady-state conditions, was calculated by

(2) $$P=\frac{273.15}{76}\frac{V\cdot l}{A\;\cdot T\cdot \;p_{0}}\frac{dp(t)}{dt}$$

where V, cm^3^ (STP), is the volume of the low-pressure compartment, *l*, cm, the membrane thickness, A, cm^2^, the effective surface area of the membrane, T, K, the working temperature, *p*_0_, bar, the feed gas pressure, dp(t)/dt, mbar/s, the slope of the straight line. The relative error of this calculation procedure was below 10%.

The extrapolation of the straight line of the time-lag graph allows for determining the time lag, θ, necessary to reach the steady state, from which the value of the diffusivity coefficient can be determined as

(3) $$D=\frac{l^{2}}{6\theta }$$

The solubility coefficient, S, is thus calculated indirectly, by the solution–diffusion model relationship between permeability and diffusivity coefficients [53]

(4) $$S=\frac{P}{D}$$

The ideal selectivity of a membrane for a gas pair separation A/B is usually determined by the ratio between the fast and slow permeabilities of the gases A and B,

(5) $$\alpha_{A/B}=\frac{P_{A}}{P_{B}}$$

Mixed gas separation of CO_2_:CH_4_ 50:50 *v*/*v*% mixtures was performed in a homemade bench-scale separation plant built at the UC [54]. The feed pressure was set at 4 bars and the composition of the feed was set by Kofloc mass flow controllers. The permeate composition was measured by an IR gas analyzer (BIOGAS 5000, Fonotest, Madrid, Spain), and the permeate flow rate by a bubble flowmeter.

## 3. Results

### 3.1. Characterization of Materials and Membranes

#### 3.1.1. Physico-Chemical and Morphological Characterization

**Figure 2:**
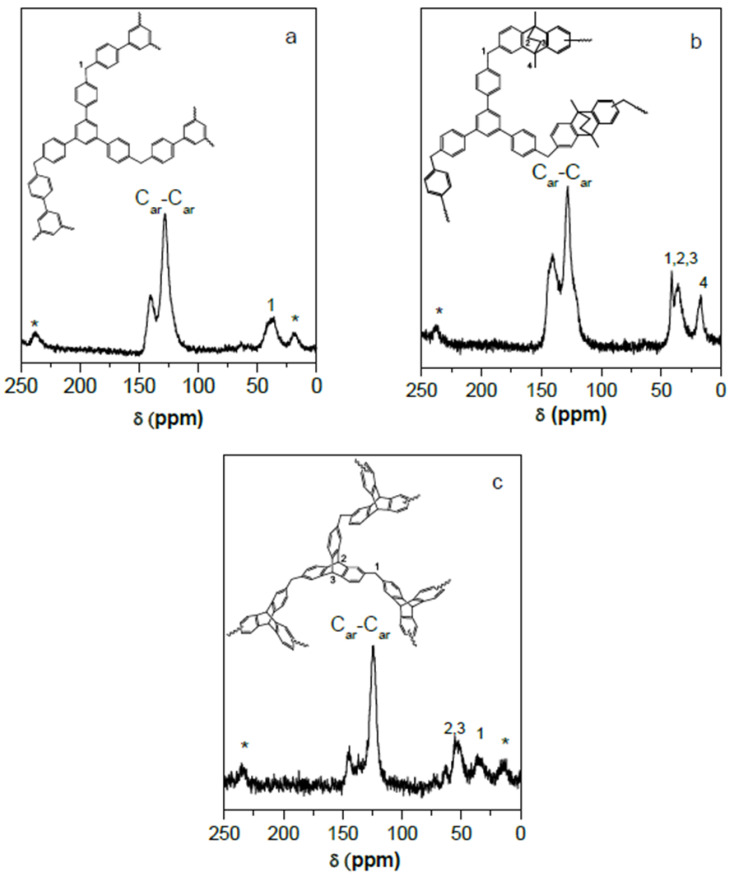
Solid-state CP/MAS ^13^C NMR spectra of (**a**) POP6, (**b**) POP3, and (**c**) POP1. Asterisks denote spinning side bands.

The POPs synthesized in this work are hyper-crosslinked materials, and therefore insoluble in organic solvents, hindering their characterization. They were identified by solid NMR and FTIR spectroscopies, although FTIR could not confirm the chemical structure due to the low intensity of the absorption bands attributed to the extreme rigidity and hardness of the material. Figure 2 shows the CP/MAS ^13^C NMR spectra of some of these materials. In general, all POPs presented wide bands that could be assigned to aromatic carbons in the interval between 110 and 150 PPM, as well as the band associated with the CH_2_ bridges around 40 ppm.

The porosity of the POPs was evaluated by their skeletal density and the adsorption/desorption isotherms of N_2_ at 77 K. Results are summarized in Table 4. The textural properties of the POPs reveal the large surface BET areas of these materials, even compared with analogous materials, which fall below 900 m^2^/g. POP1, derived from triptycene-DMM, presented the lowest microporosity, and POP6, from triptycene-DMM-biphenyl, presented the highest.

**Figure 3:**
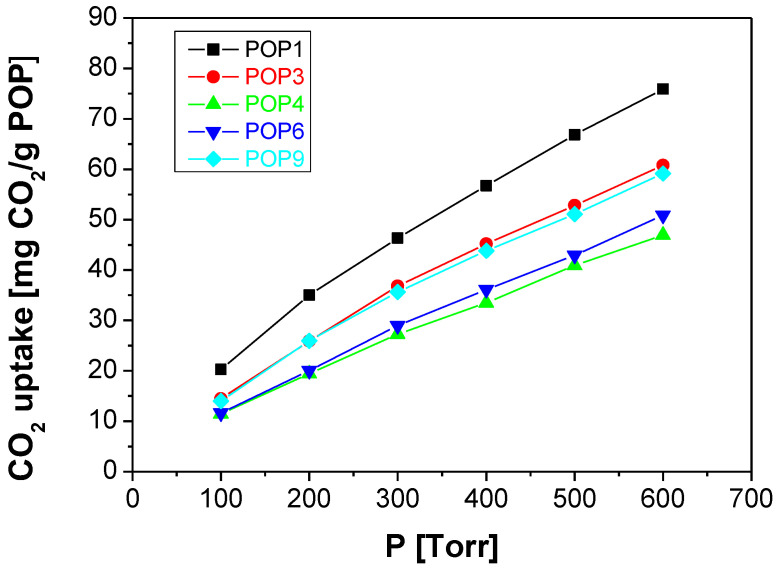
CO_2_ uptake at 298 K of the POPs used as fillers in this work.

The porosity of the POPs determines the CO_2_ affinity and thereby their use in gas separation. Figure 3 shows the CO_2_ uptake measured at 298 K for the POP fillers studied in this work as MMM fillers. The CO_2_ uptake ranges from 40 to 70 mg CO_2_/g, in the order POP4 < POP6 < POP9 < POP3 < POP1. Wang et al. reported values of CO_2_ uptake up to 45 mg/g and 23 mg/g for the freshly made and NH_2_-functionalized HOF-21 hydrogen organic frameworks [25]. POP2, synthesized from triarylamines linked by 1,4-diethynyl phenyl, provided a CO_2_ uptake of up to 176 mg CO_2_/g, on account of its high porosity, although the micropore volume was not provided [56]. Surprisingly, the POP with the highest CO_2_ uptake value was the one with the lowest microporosity and the simplest structure, POP1, and since these values agreed with those reported for SNW-1 by Gao et al. [23], they were attributed to the fact that small pores in the filler material benefited CO_2_ affinity, and thus the selective separation of CO_2_ (0.33 nm) through those pores.

**Figure 4:**
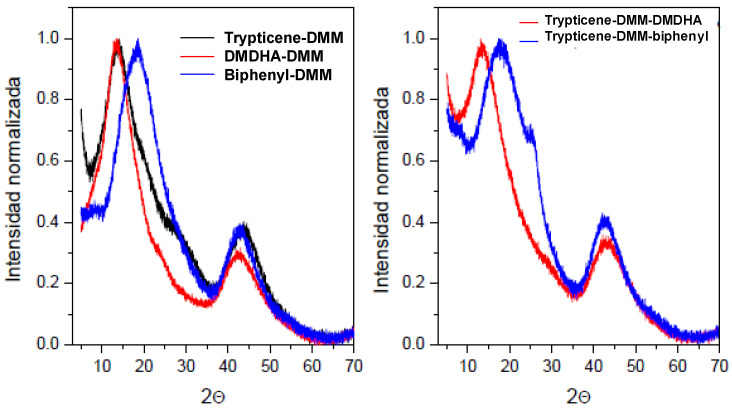
Wide-angle X-ray diffractograms of POP-1, POP-3, and POP-4 (**left**) and POP-6 and POP-9 (**right**), normalized against the maximum intensity.

**Figure 5:**
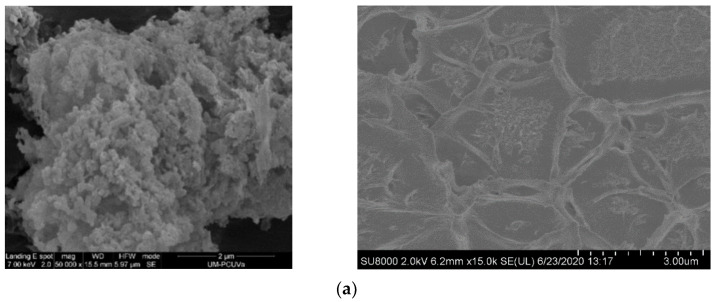

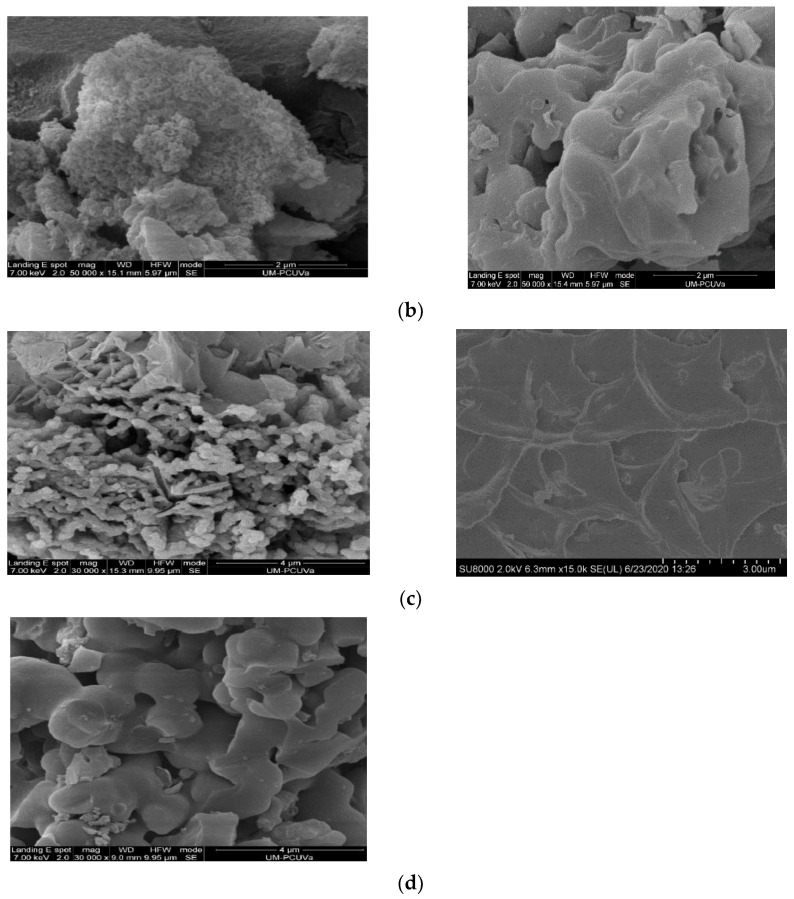
SEM of several POP filler particles are shown in the left, with the cross section of their corresponding MMMs in the right: (**a**) POP1, (**b**) POP3, (**c**) POP4, and (**d**) POP6, respectively.

For these results with the amorphous morphology of the materials, the WAXS spectra of the POPs are represented in Figure 5. All of them show a certain regularity in their chain packing, as reflected by the presence of two well-defined maxima around 2θ values 15° and 45°, respectively. Figure 4 (left) presents the diffractograms of the POPs synthesized from a single triaromatic monomer, as triptycene, or diaromatic, as DMDHA and biphenyl. Comparing all diffractograms, the maximum intensity peak in biphenyl-DMM appears at higher angle values, thus the maximum shifts from 13.6° to 18.5°, which, applying Bragg’s law, corresponds to the average preferential distances, d, of 6.49 Å and 4.79 Å, respectively. This result can be correlated with the effectiveness of the chain packing observed in POP-4, presumably due to the linear structure of biphenyl, against the non-linear 3D structure of triptycene and DMDHA, inducing the highest regularity in the network [18]. The same behavior is observed in the diffractograms of the right in Figure 5, for POP6 and POP9, derived from triptycene-DMM-DMDHA and triptycene-DMM-biphenyl, respectively.

More of the amorphous nature of the POPs and their loading into several MMM in Matrimid is discerned in the SEM images in Figure 5. The left column shows the nature of the POP particles, and the differences between POP1 (top-row), POP3, POP4 (third row), and POP6 (last row), whose porous structure started deteriorating under the electron beam upon observation. The cross sections of POP-1/Matrimid and POP4/Matrimid MMMs are also disclosed on the right column to observe the apparent absence of defects between the particles and the polyimide matrix. Thereby, we can affirm that closely compatible and defect-free membranes have been obtained.

**Figure 6:**
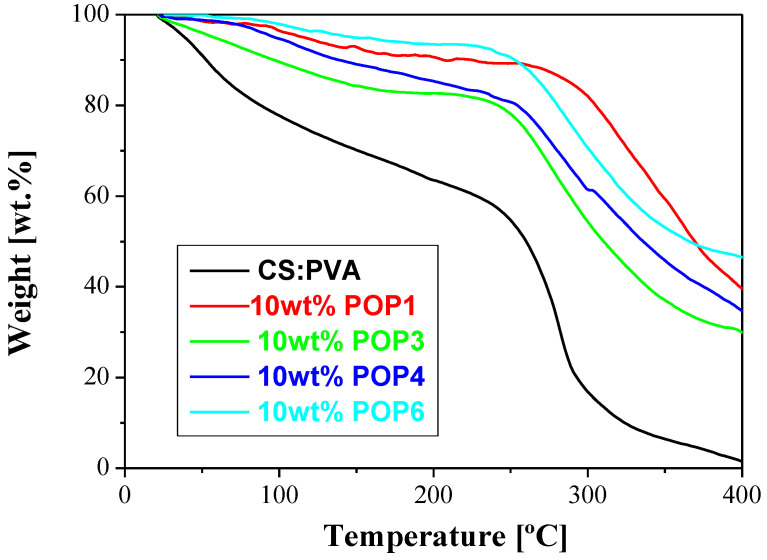
Thermal gravimetric analyses of the POP/CS:PVA MMMs.

Thermogravimetric analysis curves (TGA) were obtained for both POP fillers and POP-based MMMs under air and N_2_ to evaluate the differences in thermal stability of the material in each case. The TGA curves of water-swollen susceptible membranes in N_2_ allow analyzing the thermal stability of the membranes as well as quantifying the structural water, which may influence CO_2_ transport and separation [52,57]. Figure 6 compares a pristine CS:PVA membrane with CS:PVA membranes filled with 10 wt.% POP as a function of the type of POP. This gives an idea of whether the filler material in the hydrophilic biopolymeric matrix is homogeneously dispersed. There are two major steps, the first one below 100 °C and the second one above 200 °C (the glass transition temperature of pure chitosan is acknowledged to be at 203–206 °C [58]).

**Figure 7:**
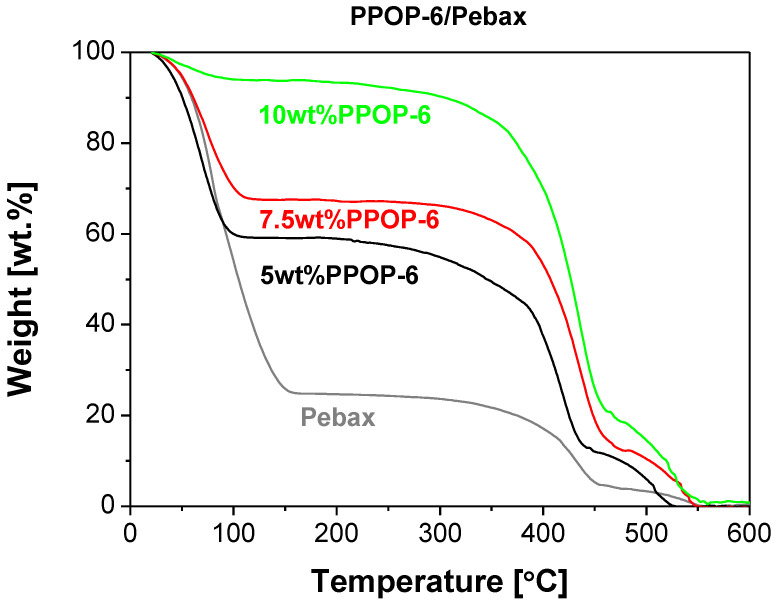
Thermal gravimetric analyses of the POP-6/Pebax MMM as a function of filler loading.

The bound water content in hydrophilic membranes was determined according to Franck-Lacaze et al. [52] using Equation (6). This water content (WC) was estimated from the mass samples; m_1_ and m_2_, measured at T_1_ and T_2_, taken as the minimum observed between the two peaks of the differential spectrum (one for water loss, one for polymer degradation), respectively, which were identified as the main weight losses observed for the CS:PVA membrane in Figure 7.

(6) $$WC\left(\%\right)=100\times \left(1-\frac{m_{1}}{m_{2}}\right)$$

As an example, Figure 7 represents the TGA analyses under N_2_ flow of the POP-6/Pebax membranes at different filler loadings. The largest weight loss due to dehydration occurred below 150 °C for pristine Pebax, and 100 °C for the POP-loaded MMMs, which accounted for the increased hydrophobicity of the POP fillers even after plasma air-treatment. Pyrolysis decomposition occurred between 300 and 450 °C, in agreement with literature [59].

The bound water content can be compared with the water uptake measured by comparing the wet and dry membranes, measured before and after the gas permeation/separation runs, as

(7) $$WU\;\left(\%\right)=\frac{w_{wet}-w_{dry}}{w_{dry}}\times 100,$$

which is also a measure of the swelling of the membranes. The values of total water absorption (WU) and bound water content (WC) were very similar for the CS:PVA membranes, which accounted for the effect of membrane synthesis and POP characteristics on the mechanical robustness of the CS:PVA-based membranes [48]. The swelling of the Pebax-based membranes was so extreme that the WU gave values well over 100%, so the WC values were taken instead in Table 5 for the estimation of the porosity, i.e., void fraction used to estimate the true volume fraction of the dispersed filler within the polymer matrix, as in previous works [50]

(8) $$\emptyset_{v}=\frac{w_{wet}-w_{dry}}{\rho_{w}}+\frac{w_{dry}}{\rho_{dry}},$$

The disparities between the true volume fraction and the nominal mass weight fraction of the filler in the membrane are due to the differences between measured density and the theoretical density values of the MMMs using the additive approach from the densities of their components. These differences have been justified by the presence of non-idealities in the interface between the porous fillers and the polymer chains, because of the partial occupation of the pores by the latter [16,50,61]. Rodriguez-Jardón et al. also observed that the calculated densities were slightly lower than the experimental ones on knitted aryl porous polymer-filled polycarbonate MMMs [28]. The higher densities obtained in the balance weighing of the samples confirmed that the pores of the fillers in the MMMs may be partially occupied by the polymer chains.

#### 3.1.2. Gas Separation and Separation Characterization

The gas separation performance was focused on the separation of CO_2_/CH_4_ mixtures. The average data of reproducible runs are collected in Table 6, as a function of polymer type, filler type, and membrane thickness. Gas permeation measurement type is indicated since Matrimid membranes were analyzed at ICTP regarding single gas permeation of CO_2_ and CH_4_, in comparison with previous work on other POP-filled Matrimid membranes [37]. POP1 decreased the CO_2_/CH_4_ ideal selectivity of pure Matrimid membranes while increasing the CO_2_ permeability. The more complex porous network of POP4 was able to maintain the Matrimid CO_2_/CH_4_ selectivity while increasing the CO_2_ permeability almost as much, which accounts for the compatibility observed earlier by SEM.

The performance of Pebax- and CS:PVA-based membranes was measured using a 50:50 (*v*/*v*%) CO_2_:CH_4_ mixed gas feed at the bench scale separation plant built at UC [54]. The results are summarized in Table 6. In general, it can be observed that the CO_2_ permeability of Pebax membranes was also influenced by the type of POP added to the Pebax matrix, in the order POP6 > POP1 > POP4 > POP3, while the CO_2_/CH_4_ selectivity was maintained in the same order of magnitude (between 10 and 16) except for the POP1/Pebax MMM, which decreased with increasing filler loading, as observed for POP-6. The 20 wt.% loading of the Matrimid matrix did not increase the selective performance of this polyimide. As for the new biopolymer-based CS:PVA-based MMMs, the CO_2_ permeability also increased in the order POP6 > POP4 > POP3 > POP1, which can be correlated with the hydrophilic nature of the CS:PVA matrix governing the interaction with the porous organic particles, since selectivity decreased in reverse order. Thus, the 10 wt.% POP3/CS:PVA membranes showed lower selectivity than the 5 wt.% POP3/CS:PVA membrane.

**Figure 8:**
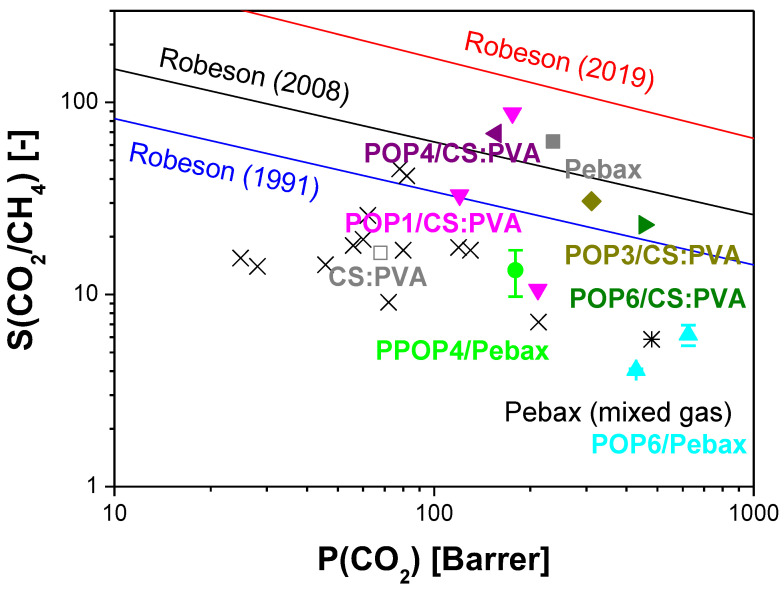
Robeson upper bound for CO_2_/CH_4_ separation. The cross points correspond to values of single gas permeation through pristine Pebax membranes in the literature. The double cross points are the values reported for the separation of CO_2_/N_2_/CH_4_ ternary mixture by Montes de Luna et al. [64]. The color points are the values of mixed gas separation performance for the POP/Pebax and POP/CS:PVA mixed matrix membranes measured in this work.

The selectivity performance of the POP-filled MMMs for CO_2_/CH_4_ separation is illustrated together with the Robeson plots in Figure 8. The Robeson plots indicated in the graph are those reported by Robeson himself in 1991 [62] for the first polymer films with gas permeation data and the updated ones in 2008 with new polymer advancements and gas pair mixtures [63]. The line named “Robeson (2019)” is the one updated by Comesaña-Gandara et al. to consider specifically the separation of CO_2_ from other gases typical in industrial effluent [10]. We can observe that POP1- and POP4-based CS:PVA MMMs surpass the state-of-the-art in polymer membranes for the separation of CO_2_/CH_4_ gas pairs, while the Pebax membranes prepared in this work do not. This result may be attributed to the difficulty of making Pebax membranes with these types of filler that need to be functionalized further for compatibility. Wang et al. [25] functionalized their HOF-21 with NH_2_ groups to obtain a defect-free membrane enabling the evaluation of the influence of the hydrogen organic framework in the Pebax matrix.

The separation factor of the gas mixture presented in Table 6 for CS:PVA-based MMMs was calculated from the concentration of slow and fast gas permeating molecules in the feed and permeate streams respectively. We can observe that the separation factor agreed with the ideal selectivity calculated by Equation (5), which can be assigned to good compatibility between the CS:PVA continuous matrix and the POP fillers. Clearly, the plasma treatment of the POP particles was not enough to improve their compatibility with Pebax, and non-idealities occurred that will be analyzed below.

### 3.2. Mechanism of Transport through MMMs

An investigation of the overall permeability behavior of new mixed matrix membranes involving a dispersed phase (filler) or a continuous phase (polymer matrix) is essential to obtain new materials with improved properties. The main modeling parameters are: (1) the single gas permeability coefficients of the component of the feed mixture (assuming they are independent of the concentration of the permeating species in a mixed gas environment), (2) the composition of the MMM, expressed as the true volume fraction of the dispersed filler, and (3) the shape and arrangement of the dispersed particles in the MMM.

The most used phenomenological model to describe MMM transport properties is the Maxwell equation that describes the overall permeability through a composite medium of a highly diluted dispersion of congruent spheres in a continuous phase, where interparticle distances are large enough to ensure that the permeant flow pattern around each particle is not disturbed by the presence of the others. This equation can be written as

(9) $$P_{MMM}=P_{c}\left[\frac{P_{d}+2P_{c}-2\emptyset_{d}\left(P_{d}-P_{c}\right)}{P_{d}+2P_{c}+\emptyset_{d}\left(P_{d}-P_{c}\right)}\right],$$

where P_MMM_ is the effective permeability of the mixed matrix membrane material, P_c_ is the permeability of the continuous polymer phase, measured experimentally on the pristine polymer membrane, and P_d_ is the permeability of the dispersed filler phase, respectively. Ø_d_ is the true volume fraction of the dispersed filler (Table 4). Since MMMs are often made from novel filler materials never tested as membranes before, as is the case with POPs, Equation (9) has been rearranged as follows [65]:

(10) $$P_{MMM}=P_{c}\left[1+\frac{3\emptyset_{d}}{\left(\alpha +2\right)\left(\alpha -1\right)-\emptyset_{d}}\right]$$

where $\alpha \;*=P_{d}/P_{c}$ is an adjustable parameter. Rodriguez-Jardón et al. [28] simplified it further for porous organic fillers by defining a new parameter β, accounting for the reduced permeation polarizability observed in polyimide-like polymer matrices, and only depending on gas permeabilities.

(11) $$P_{MMM}=P_{c}\left[\frac{2\left(1-\emptyset_{d}\right)+\alpha \left(1+2\emptyset_{d}\right)}{\left(2+\emptyset_{d}\right)+\alpha \left(1-\emptyset_{d}\right)}\right]=P_{c}\frac{1+{2\emptyset }_{d}}{1-\beta \emptyset_{d}}$$

where

(12) $$\beta =\frac{\alpha -1}{\alpha +2}=\frac{P_{d}-P_{c}}{P_{d}+2P_{c}}$$

**Figure 9:**
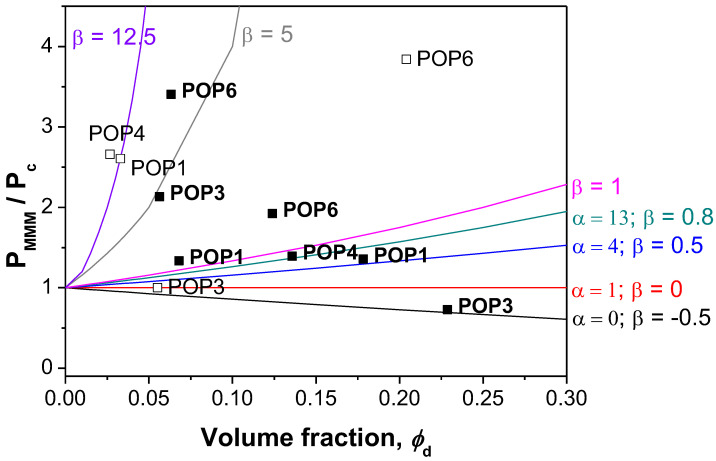
Comparison between the experimental data for CO_2_ permeability through POP-filled Pebax and CS:PVA MMMs and the Maxwell model predictions using Equations (10) and (11).

Figure 9 uses these analyses to evaluate the CO_2_ permeability of the POP membranes prepared in this work, with parameter β ranging from −1 to 12.5. Literature trying to describe the gas transport through MMMs varied this parameter between −0.5 and 1.0 to represent non-permeable and wholly permeable fillers. The value of β = 0 meant a value for α having equal permeability in both continuous and dispersed phases. As with them, our POPs are porous and thus totally permeable so β should be closer to 1.

As expected, since the Maxwell model equation assumed the diluted dispersion of spherical particles, the performance of the higher-porosity fillers like POP9- and POP6-based membranes deviated from the description of this model. A first evaluation of the prediction capability of the gas transport properties through a new MMM involved evaluating the limits of the Maxwell equation. The minimum and maximum limits of the Maxwell equation have also been expressed as a function of the membrane composition and the differences in permeability through the continuous and dispersed phases, by considering a series mechanism of transport through the dispersed and continuous phases as

(13) $$P_{MMM}=\frac{P_{c}P_{d}}{\left(1-\emptyset_{d}\right)P_{d}+\emptyset_{dP_{c}}}=P_{c}{\left[1+\emptyset_{d}\left(\frac{1}{\alpha }-1\right)\right]}^{-1}$$

and the maximum value is assumed when both phases contribute in parallel to the flow direction,

(14) $$P_{MMM}=\emptyset_{d}P_{d}+\left(1-\emptyset_{d}\right)P_{c}=P_{c}\left[1+\emptyset_{d}\left(\alpha -1\right)\right]$$

The accuracy between the experimental values (Table 6) and the values predicted with Equations (9), (13) and (14) has been compared in terms of the percentage average absolute error (AARE) and collected in Table 7.

**Figure 10:**
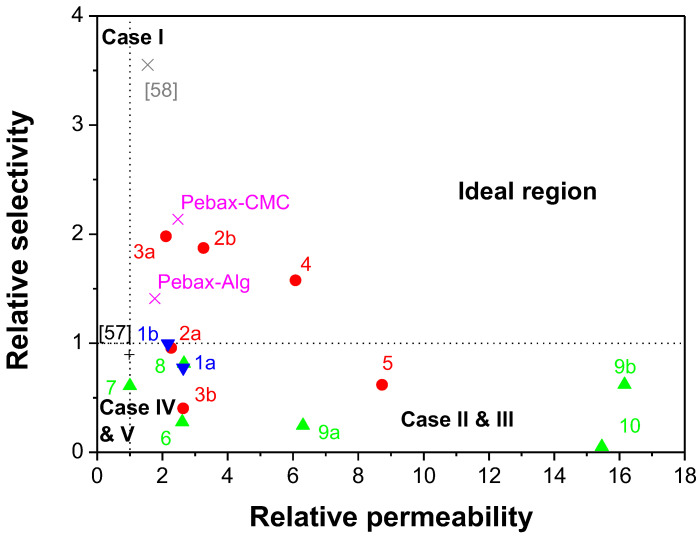
Relative selectivity and permeability of the different POP-filled MMMs in Pebax and CS:PVA matrices as a function of the different morphology cases identified in MMMs. (1a) POP1/Matrimid; (1b) POP-4/Matrimid; (2a) 5%POP1/CS:PVA; (2b) 10 wt% POP-1/CS:PVA; (3a) POP3/CS:PVA; (3b) 10 wt% POP3/CS:PVA; (4) 10 wt% POP4/CS:PVA; (5) 10 wt% POP6/CS.PVA; (6) POP1/Pebax; (7) POP3/Pebax; (8) POP4/Pebax; (9a) 16 wt.% POP6/Pebax; (9b) 32 wt.% POP6/Pebax; (10) POP9/Pebax. Also represented in the figure, CS, swollen [67], CS, TMC [66], Pebax-Alg, and Pebax-CMC [68].

The correlation between overall transport properties and the structure of the interface plays an important role in the development of composite membrane material. Four major cases explaining this correlation when porous fillers are used have been a matter of discussion for a long time [16]. Figure 10 collects the data in Table 5 and Figure 8 in terms of these cases:

- Case 1 corresponds to an ideal behavior or perfect contact between the polymer matrix and the filler.
- Cases II and III are characterized by voids at the interface, causing an increase in permeability without large changes in selectivity, in comparison with pure polymer membranes. In Case II, the effective void thickness is of the order of magnitude of the gas penetrant molecules. Most of the Pebax-based MMMs belong to this range.
- Cases IV and V, where a rigidified polymer region is estimated around the filler causing reductions in permeability and a slight increase in the selectivity of the MMMs in comparison with the pure polymer membrane. Unsurprisingly, the Matrimid MMMs fall into these categories, and are attributed to the rigidified polyimide structure of Matrimid.

We observe that the POP/Pebax MMMs fall within the category of Cases II and III, on account of the high permeability of Pebax and the porosity of the POPs, especially POP6, imparted to the polymer matrix. Matrimid-based membranes, unsurprisingly, fall close to Cases IV and V, on account of the rigid polyimide matrix of Matrimid. The POP/CS:PVA MMMs fall mid-way, which may be attributed to the semi-crystalline nature of biopolymers and the compatibility with organic fillers with high porosity compatible with the biopolymer functional groups, which may expect some penetration of polymer chains with the POP structure and thus the slight decrease in permeability and increase in selectivity observed for the most porous POPs (POP3, POP6). The tunable hydrophilicity of biopolymers can alter the transport mechanism through the polymer matrix from solution diffusion in TMC-crosslinked chitosan [66], to facilitate transport in swollen chitosan membranes [67]. Pebax could also be blended with biopolymers in this way, as Salestan et al. [68] have reported recently using small loadings of alginate and CMC.

## 4. Discussion

Comparison of the experimental data with the prediction of Maxwell’s ideal model confirmed the presence of non-idealities at the interface of POPs and different polymeric matrices [69].

The CO_2_ permeability values of the CS:PVA and Pebax-based MMMs are underestimated by the Maxwell model, these values being closer to the maximum limit determined by the parallel version of Equation (14). In fact, the Matrimid-based MMMs do not converge using Equation (13), which establishes the minimum permeability predicted from the Maxwell model, probably due to the low permeability values through this polyimide. Another interesting feature observed in these POP-based MMMs that differs from other materials is that the greater the selectivity that the filler material imparts to the heterogeneous MMM, the greater its deviation from the ideal model equation. Figure 10 also highlights that these deviations are more relevant in Pebax-based MMMs, due to the higher porosity of some of the POPs (as in the case of POP9) and a higher amount of filler particle loading (POP6), as observed experimentally. It seems evident that the plasma treatment of the particles was not sufficient to improve the compatibility of polymer and fillers when high-porosity POPs and high polymer network loadings are employed. It is commonly accepted that at high loadings, the Bruggeman model gives a better result, since it approximates the case where the difference in permeability of the dispersed and continuous phases decreases, making α* = 1.

Different phenomenological expressions have been described in the literature to describe the effect of porous organic fillers in MMMs. One of the seminal works was that of Vu et al. [16], in which it was observed that the ideal Maxwell model provided a poor prediction of the observed permeability through MMMs made by Ultem or Matrimid matrices and carbon molecular sieve (CMS) fillers (these materials possess lower permeability values than the Pebax and CS:PVA MMMs prepared in this work). When the predicted permeability is lower than the experimentally obtained permeability, this is generally attributed to particle agglomeration, which causes gas molecules to diffuse preferentially through the particle channels rather than the hypothetical uniform dispersion of the complex MMM system [70] since the ideal Maxwell model does not account for the non-ideal morphologies discussed in Figure 10. Table 7 reflects the mean absolute experimental errors of the CO_2_ and CH_4_ permeability values predicted by Equations (9), (13) and (14), respectively, where the permeability value through CMS reported in literature is used as a reference for the permeability of the dispersed phase, P_d_, in these equations [16]. It can be seen that the gas permeability through the Matrimid-based MMMs is best predicted by the lower bound of the ideal Maxwell model, represented by Equation (13), while the more permeable hydrophilic MMMs based on Pebax and CS:PVA polymers approach the limit represented by Equation (14). The exception to this behavior corresponded to the POP-6-filled MMMs that deviated from the ideal morphology described by the Maxwell model, which can be attributed to the higher porosity afforded by this POP6 to the heterogeneous structure of the MMMs when compared to the other MMMs in this work. These observations agree with data from the literature for other organic cage fillers and PEEK-WC, compared with Matrimid, in MMMs for gas separation [21].

Thus, in this work, modifications of the ideal Maxwell model equation have been evaluated by applying Equation (9) twice, to account for the thickness of the stiffened or empty region between the dispersed porous particle and the continuous polymeric matrix (interface), and the chain immobilization factor that accounts for the decrease in permeability in the vicinity of the particle if stiffening occurs (Cases IV and V in Figure 10) as observed in the literature for CMSs in Matrimid [16]. This modification can be improved to account for the non-ideal pore-blocking behavior that occurs when polymeric chains penetrate porous fillers (Cases II and III in Figure 10), which could explain, in some cases, the increased permeability of the MMM compared to the original pristine polymeric membrane [38]. Thus, Gheimasi et al. included partial pore blocking to predict CO_2_/CH_4_ separation through CMS-filled MMMs [68]. However, in the modifications of phenomenological MMM transport models applied in carbon molecular sieve-filled MMMs, they only optimized the form factor, n [69], assuming n = 1/3 as in Equation (9), as Nasir et al. [70] did to fit their carbon molecular sieve-filled PES MMMs behavior regarding CO_2_ separation. Applying Equation (9) twice as a function of two parameters describing the interface/void (interface) distance between the dispersed and continuous phases. For instance, the interface distance values of 0.54 and 1.06 μm, between the POP6 particles and the CS.PVA and Pebax continuous matrices, respectively, and immobilization factors of 0.14 and 0.10, and 0.10 and 0.57, for CO_2_ and CH_4_, in the CS:PVA and Pebax continuous matrices, respectively, gave AARE of the gas permeability prediction through the MMMs lower than 0. 001%. These values agree with the results obtained previously for ionic liquid/chitosan (IL-CS) MMMs filled with porous ZIF-8 and HKUST-1 nanoparticles [50].

These behavioral observations are attributed to the high CO_2_ uptake and differences in the porosity of the POP fillers, with POP6 being larger than the others, and to the dependence of diffusivity on permeability through rigid Matrimid polyimide membranes [21,24], and to the gas solubility that is probably facilitating CO_2_ transport through such hydrophilic polymers as Pebax and CS:PVA [28].

## 5. Conclusions

A new set of mixed matrix membranes was prepared by blending conventional polymers and several porous organic hyper-crosslinked polymers (POPs) as fillers, which were studied in CO_2_/CH_4_ separation. The employed polymer matrixes were Matrimid and Pebax, and a biopolymer base made from chitosan and polyvinyl alcohol. The gas separation performance was measured in terms of single gas permeability and mixed gas CO_2_/CH_2_ (50:50 *v*/*v*%) separation. The compatibility of the POP particles into the polymer matrix was improved by POP plasma treatment before making the blend, for the hydrophilic Pebax and CS:PVA biopolymer. It was observed that the compatibility of the POP particles into the polymer matrices, for the hydrophilic Pebax and CS:PVA biopolymer, was improved by POP plasma treatment before making the blend. The compatibility effect between the porous fillers and matrices onto gas transport was studied by using the Maxwell model as a function of the gas permeability of the pure polymers, the porosity and composition of the fillers, and the composition of the MMMs.

It was observed that the materials could be described by the simple Maxwell model, except in the case of the highly porous POP-derived membranes, where the increased porosity generated non-idealities in the transport mechanism. These anomalous situations should be further explored by considering other issues such as the free volume of the material and the facilitated contribution to the transport mechanism that could occur in the membrane. The performance of bio-based CS.PVA membranes as gas separation membranes approaches the performance of hydrophilic Pebax membranes, which makes these CS.PVA membranes present potential for application in commercial membranes, once the understanding of the influences of the mass transport mechanism is clarified by careful determination of structure-property relationships.to accelerate the development of sustainable membranes for different applications by widening the range of materials available for membrane fabrication under criteria within the circular economy.

## Figures and Tables

**Table 2:** The molar composition of monomers and reactants used in the syntheses of the POP fillers in this work.

POP	Triptycene	135-TPB ^1^	Biphenyl	DMDHA ^2^	DMM ^3^	FeCl_3_
POP1	1	-	-	-	3	3
POP3	1	-	-	0.67	4.33	4.33
POP4	1	-	0.67	-	4.33	4.33
POP6	-	1	-	-	3	3
POP9	-	1	-	0.67	4.33	4.33

^1^ 135-TPB = 1,3,5-triphenylbenzen; ^2^ DMDHA = 9,10-dihydro-9,10-dimethyl-9,10-ethanoanthracene; ^3^ DMM = Dimethoxymethane.

**Table 3:** Properties of the polymers used as continuous matrices for MMM preparation.

Property	Matrimid 5218	Pebax MH 1657	Chitosan
Chemical structure	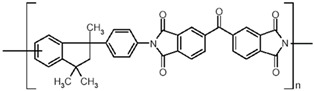	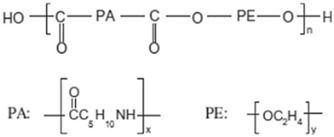	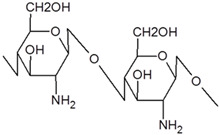
Tg (°C)	317 [39]; 308 [40]	−53	203 [41]
Density (g cm^−3^)	1.225 [42]	1.14	0.942 [43]
Melting point (°C)	>300 [40]	204 ^1^	270 [44]
Estimated fractional free volume	0.21 [45]	0.143 [46]	0.228 [47]

^1^ Data provided by the supplier.

**Table 4:** Textural properties of the POP fillers studied in this work.

Type of POP	Skeletal Density (g/cm^3^)	S_BET_ (m^2^/g)	V_TOTAL_ (cm^3^/g)	V_MICRO_ (cm^3^/g) *	Reference
POP1	1.2624	1538	1.281	0.314	This work
POP3	1.2051	1596	1.394	0.293	This work
POP4	1.1994	1318	0.727	0.368	This work
POP6	1.2014	1638	0.964	0.450	This work
POP9	1.2288	1525	1.606	0.325	This work
POP2	1.33	781	0.554	NA	[55]
KAP (2Ph-NO_2_)	1.618	605	0.313	NA	[28]
KAP (2Ph-CH_2_NH_2_)	1.459	617	0.282	NA	
SNW-1	NA	821	NA	0.26	[36]
TRPI (135TRP-DAFO)	1.113	806	0.42	0.24	[37]

NA = Not Available. * Volume determined at p/p_0_ = 0.9768.

**Table 5:** Some morphological properties of the MMMs.

Membrane	Filler wt. Fraction	Thickness (cm)	Density (g/cm^3^)	WU (%)	WC (%)	T_d_ (°C)	Porosity (%)	Volume Fraction, ø*_d_*
Matrimid [37]	0	0.005	1.223 [60]	NA	NA	NA	16.7 [60]	0
POP1/Matrimid	0.20	0.005	1.232		NA			0.195
POP4/Matrimid	0.20	0.005			NA			0.203
Pebax	0	0.01102	-	(*)	58	290	-	0
POP1/Pebax	0.05	0.00772	1.124	(*)	34	172	27	0.033
POP3/Pebax	0.05	0.00785	1.225	(*)	59	226	42	0.028
POP4/Pebax	0.05	0.00818	1.289	(*)	60	222	44	0.027
POP6/Pebax	0.16	0.0189	1.009	50	53.7	377	40	0.091
	0.32	0.0250	1.240	64		219	34	0.204
POP9/Pebax	0.10	0.0934	1.240	64	50.8	219	40	0.052
CS:PVA	0	0.016	1.749	39.80 ± 1.26		131	41	0
POP1/CS:PVA	0.05	0.0147	1.349	47.88	33	171	39	0.041
	0.10	0.0098	1.782		32	226	48	0.070
POP3/CS:PVA	0.05	0.0097	2.147	37.2	40	171	44	0.039
	0.10	0.0136	1.305		23	172	18	0.111
POP4/CS:PVA	0.10	0.01185	1.389	18	23	172	20	0.111
POP6/CS:PVA	0.10	0.0133	0.850	14.5	17	242	11	0.124

(*) Values over 100% have been removed.

**Table 6:** CO_2_ permeability and CO_2_/CH_4_ selectivities and separation factors of the POP-based MMMs studied in this work. Only selective membrane materials are included.

Polymer Matrix	POP, Filler Loading	Thickness (cm)	P(CO_2_) (Barrer) ^(a)^	P(CH_4_) (Barrer) ^(a)^	α(CO_2_/CH_4_)	S.F. (CO_2_/CH_4_)
Matrimid ^(b)^	0	0.005	7.84	0.186	42	-
	POP1, 20 wt.%	0.005	20.68	0.632	37	-
	POP4, 20 wt.%	0.005	17.0	0.400	42	-
Pebax ^(c)^	0	0.011	67.95 ± 13.51	4.132	16.44 ±0.31	11.78
	POP1, 5 wt.%	0.077	176.95 ± 5.17	38.73	4.57 ± 0.32	3.98
	POP3, 5 wt.%	0.0078	67.95 ± 13.50	6.79	10.01 ± 3.25	-
	POP4, 5 wt.%	0.0082	180.68 ± 90.54	13.50	13.38 ± 3.64	12.0
	POP6, 16 wt.%	0.0189	1098	107.82	10.18	7.88
	POP6, 32 wt.%	0.0255	428 ± 15.5	106.20	4.034 ± 0.02	3.21 ± 0.04
	POP9, 8.3 wt.%	0.00632	1050	1282	0.82	0.85
CS:PVA ^(c)^	0	0.01605	51.99	1.55	33.64	31.19
	POP1, 5 wt%.	0.0147	66.15	2.06	32.14	31.43
	POP3, 5 wt.%	0.0097	109.80	1.65	66.59	27.50
	POP3, 10 wt.%	0.0136	36.86	2.73	13.50	13.00
	POP4, 10 wt.%	0.0118	62.81	1.19	53.00	62.5
	POP6, 10 wt.%	0.0133	453.80	20.75	21.80	17.30

^(a)^ 1 Barrer = 10^−10^ cm^3^ (STP) cm cm^−2^ s^−1^ cmHg^−1^; ^(b)^ time-lag experiments performed at the University of Valladolid, as in [37]; ^(c)^ mixed gas separation experiments with a 50:50 (*v*/*v*%) CO_2_:CH_4_ feed mixture at the University of Cantabria, as in [54].

**Table 7:** Percentage (%) of average absolute relative error (AARE) for the sum of calculated CO_2_ and CH_4_ permeation prediction for each MMM.

Continuous Matrix	Dispersed Phase	Parallel	Series	Maxwell
Matrimid	POP1	13.11	15.26	11.18
	POP4	5.12	22.26	17.12
Pebax	POP1	31.02	31.13	31.05
	POP3	0.48	0.74	4.40
	POP4	31.37	31.46	31.39
	POP6	42.6	42.8	6.42
	POP9	-	-	-
CS:PVA	POP1	10.96	10.99	10.97
	POP3	26.47	26.50	27.38
	POP4	51.09	9.43	43.55
	POP6	44.38	44.39	44.38

## Data Availability

Not applicable.
